# An “Uncrimped” SMart Stapes Prosthesis: A Cause of Late Hearing Deterioration in Otosclerosis

**DOI:** 10.1155/2012/120267

**Published:** 2012-01-31

**Authors:** Premjit S. Randhawa, Nicholas Hamilton, Antony A. Narula

**Affiliations:** Department of Otolaryngology, St Mary's Hospital, Imperial Healthcare NHS Trust, Praed Street, London W2 1NY, UK

## Abstract

*Statement of Problem*. Stapedotomy is the treatment of choice for otosclerosis. Numerous techniques and prosthesis are available to perform this procedure. Success rates of surgery vary from 17% to 80%, and revision surgery carries an increased risk of complications as well as poorer hearing outcomes. *Method of Study*. Case report. *Results*. We report the first case of uncrimping of a SMart stapes prosthesis with no lateral displacement as a cause of late failure despite successful crimping and improvement in audiological outcomes after initial surgery. *Conclusion*. The SMart stapes prosthesis is widely used and has been shown to be safe and provide good hearing outcomes. Displacement of a stapes prosthesis is the commonest cause of failure. Our case shows that deterioration of hearing thresholds can occur from uncrimping of the prosthesis with no displacement. It is important to improve our understanding of stapedotomy failure as revision procedures are associated with poorer outcomes.

## 1. Introduction

Since Shea introduced stapedotomy in 1958 as standard treatment for otosclerosis, there have been a myriad of prostheses that have been developed [1]. The selection of a particular prosthesis by individual surgeons is dictated by the ease of usage, safety and favourable hearing outcomes. An “ideal” stapes prosthesis should be easy to apply, safe to use and produces good hearing results. 

The SMart stapes piston prosthesis (Gyrus-ENT; Bartlett, TN, USA) is a newer addition to the wide range of prostheses that are available. It is composed of a nitinol-based Shepard hook and a Telfon-based piston. Nitinol is a metal alloy of nickel (45%) and titanium (55%) that allows self-crimping by heat activation [2]. This feature of Nitinol (nickel-titanium alloy) has been adopted and used for many years in medical applications such as catheters and stents across various subspecialities [3, 4]. It has been proven to be highly biocompatible and well accepted in short-and-long term implantation [5, 6].

The SMart prosthesis is marketed on the ability of its piston wire to self-fasten or crimp securely around the incus following the application of heat. This self-crimping property is reported to provide a more secure fit between the wire and the incus which in turn leads to improved transmission of sound [7]. By eliminating manual crimping and reducing the manipulation of the prosthesis in the middle ear, the SMart prosthesis should theoretically lower failure rates.

Although the safety and good hearing outcomes of the SMart stapes prosthesis are well reported, there have been reports of Nitinol causing inflammatory reactions in the middle ear mucosa and sensorineural hearing loss [8]. A number of studies have also reported lateral displacement as a cause of persistent or recurrent conductive hearing loss [9, 10]. Fibrous adhesions around the footplate and piston as well as displacement from the fenestra have also been reported [10].

We report a case of “uncrimping” with no lateral displacement of the SMart stapes prosthesis 3 years after successful application with deterioration of hearing thresholds. To our knowledge, this is the first reported case in the literature.

## 2. Case Report

**Figure 1:**
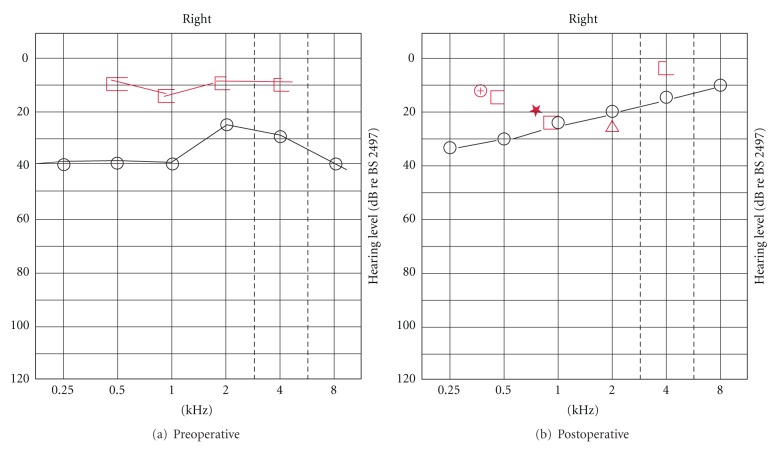
Pre- and postoperative pure-tone audiogram.

A 33-year-old lady with bilateral otosclerosis underwent a right stapedotomy via an endaural approach by the senior author using a 4.5 × 0.6 mm SMart prosthesis. There were no complications intraoperatively, and the prosthesis was successfully “crimped” with bipolar forceps (2 watts). There was good improvement in her hearing outcomes postoperatively (Figure 1).

**Figure 2:**
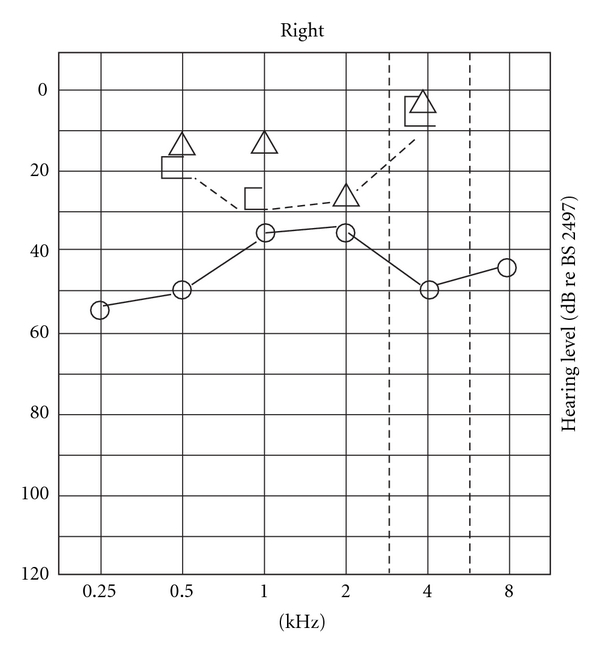
Pure-tone audiogram prior to revision surgery.

She presented 3 years later with a 2-month history of worsening hearing in her right ear. The only significant history of note was that she suffered some pain in her ear while flying some 12 months earlier. Clinical examination was unremarkable with an intact tympanic membrane. Pure-tone audiometry showed deterioration in her hearing thresholds (Figure 2). She underwent a fine-cut computed tomography (CT) scan of her temporal bones which did not reveal a cause for the deterioration in hearing thresholds. A decision was made for her to undergo a tympanotomy ± revision stapedotomy. 

**Figure 3:**
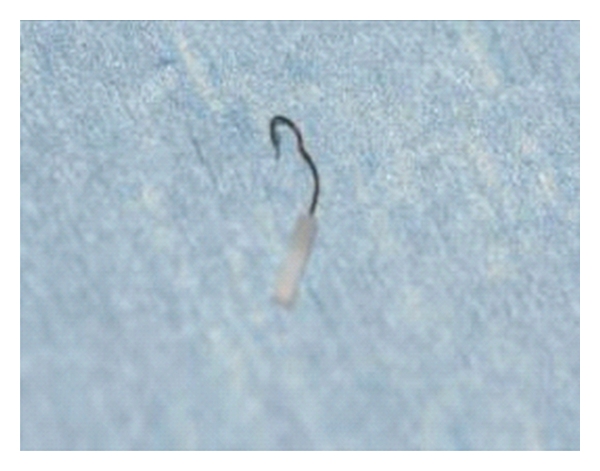
“Uncrimped” SMart prostheses.

At time of surgery, the SMart prosthesis was found to be uncrimped (Figure 3) although still “hanging” onto the long process of the incus and into the footplate. This was subsequently removed and replaced with 4.5 × 0.6 mm Schuknecht piston. Her hearing thresholds improved post-operatively with good closure of the air-bone gap.

## 3. Discussion

Success rates of stapedotomy are traditionally assessed by the closure of the air-bone gap on audiometry with a good outcome generally accepted to be within 10 dB or less, closure of the preoperative air-bone gap. Success rates of surgery vary from 17% to 80% [11]. The common causes of failure of stapedotomy include prosthesis displacement, incus necrosis, undiscovered fixation of the incus or malleus, adhesions in the middle ear, and oval window fibrosis. Revision stapedotomy procedures are associated with inferior hearing outcomes and greater comorbidity [11, 12]. 

The SMart prosthesis is one of the newer stapes prostheses available on the market, and a number of large series have shown good surgical outcomes with its usage [13, 14]. The piston exhibits shape memory whereby the application of heat leads to a phase alteration in the atomic structure resulting in the formation of a predetermined shape. In the case of a SMart prosthesis, this results in the crimping of the hook around the incus. As this crimping occurs, increased contact with the incus generates stress which acts to inhibit the memory properties and therefore stop the crimping process [15]. This should produce a snugly fitted secure prosthesis.

In our case, the prosthesis was successfully crimped at time of initial surgery with good audiological evidence of improvement. The prosthesis, however, was found to be “uncrimped” during surgery. We would not classify this as a prosthesis displacement, the commonest cause of late failure of surgery, as the piston was still in contact with the incus and the stapes footplate. The uncrimping of the prosthesis has led to poor sound transmission and hence deterioration of hearing thresholds.

It is unclear as to the reasons why this occurred in our case. There could well have been an inherent problem with the manufacturing of this individual prosthesis, whereby the ratio of titanium and nickel (55% versus 45%) deviated from the recommended ratio, leading to a prosthesis with poor shape memory that is liable to “uncrimp.” Another possibility was that the shape memory of the hook could have been lost if the alloy was exposed to excessive stress, but at no point during the procedure was this likely to have occurred as the prosthesis was heat-activated. Although there have been no reported cases of the SMart prosthesis uncrimping, there have been unreported cases which the authors are aware of, in which this problem has occurred. 

A number of publications have highlighted persistent or recurrent conductive hearing loss with the use of the SMart prosthesis. The main finding of these studies was that the prosthesis had in fact been displaced laterally, either with the hook in its closed or opened state, and no contact was present with the long process of incus (LPI) [9, 10]. Another common finding is that the prosthesis is found lying outside the fenestra [10]. In our case, contact still remained but the “uncrimped” state of the piston affected sound conduction. 

Nitinol as an alloy itself that is used in medical equipment has been proven to be highly biocompatible and well accepted in short and-long-term implantation [5, 6]. Data regarding early or long-term changes in configuration of the material itself is not readily available. 

The authors accept that there are large published series of good hearing outcomes with the SMart prosthesis; however, we feel that clinicians should be aware of the possibility of the SMart prosthesis “uncrimping,” despite being in contact with the stapes footplate and LPI, and causing late failure in stapedectomy.

## 4. Conclusion

This paper presents the first published case of a SMart stapes prosthesis that has uncrimped and caused deterioration in hearing thresholds despite still being in contact with the LPI and stapes footplate. It is important to improve our understanding of stapedotomy failure as revision procedures are associated with poorer outcomes.
